# Silkworm Gut Fiber of *Bombyx mori* as an Implantable and Biocompatible Light-Diffusing Fiber

**DOI:** 10.3390/ijms17071142

**Published:** 2016-07-16

**Authors:** Jose Luis Cenis, Salvador D. Aznar-Cervantes, Antonio Abel Lozano-Pérez, Marta Rojo, Juan Muñoz, Luis Meseguer-Olmo, Aurelio Arenas

**Affiliations:** 1 Department of Biotechnology, Instituto Murciano de Investigación y Desarrollo Agrario y Alimentario (IMIDA), Murcia 30150, Spain; josel.cenis@carm.es (J.L.C.); sdac1@um.es (S.D.A.-C.); 2 Departamento de Electromagnetismo y Electrónica, Universidad de Murcia, Murcia 30003, Spain; mrojo@um.es (M.R.); juanmu@um.es (J.M.); arenas@um.es (A.A.); 3 Biomaterials & Tissue Engineering Unit & Orthopedic Surgery Service, V. Arrixaca University Hospital, Murcia 30120, Spain; lmeseguer.doc@gmail.com; 4 Department of Health Sciences, UCAM-Catholic University of Murcia, Murcia 30107, Spain

**Keywords:** silkworm gut fiber, biocompatibility, light-diffusing optical fiber, silk fibroin

## Abstract

This work describes a new approach to the delivery of light in deeper tissues, through a silk filament that is implantable, biocompatible, and biodegradable. In the present work, silkworm gut fibers (SGFs) of *Bombyx mori* L., are made by stretching the silk glands. Morphological, structural, and optical properties of the fibers have been characterized and the stimulatory effect of red laser light diffused from the fiber was assayed in fibroblast cultures. SGFs are formed by silk fibroin (SF) mainly in a β-sheet conformation, a stable and non-soluble state in water or biological fluids. The fibers showed a high degree of transparency to visible and infrared radiation. Using a red laser (λ = 650 nm) as source, the light was efficiently diffused along the fiber wall, promoting a significant increment in the cell metabolism 5 h after the irradiation. SGFs have shown their excellent properties as light-diffusing optical fibers with a stimulatory effect on cells.

## 1. Introduction

Light has a wide array of effects on cells and living tissues [1]. Some of these effects are positive and have been developed as therapies by biomedical research. One of these therapeutic approaches based on the stimulatory effect of light on cells is low-level laser therapy (LLLT), which can stimulate a number of biological processes—mainly cell growth, proliferation, and differentiation—in a diversity of cell types [1]. This effect has been attributed to the stimulation of chromophores present in the respiratory chain in mitochondria, which results in an increment of ATP synthesis and a general enhancement of cell metabolism and proliferation [2,3,4,5]. This effect is most efficient at energy density values of 0.5 to 4 J/cm^2^ and in the visible spectrum ranging from 600 to 700 nm. Therapeutic effects of LLLT include an improvement of wound healing in skin ulcers, among many others [6,7].

A different family of applications of light is photodynamic therapy. In this case, light stimulates a photoactivated molecule that, as a consequence, forms oxygen singlets that are highly oxidant of living cells. This results in the apoptosis of cells. When close to tumor cells, this approach functions as an antitumor therapy [8,9]. Another use of light that merits mention is the field of optogenetics, where a pulse of coherent light acts on opsins. These are chromophores, present in bacteria, whose genes are inserted in neurons by transformation. The stimulation of opsins can open or close ionic channels in transformed neurons, allowing potential actions to be started or stopped [10,11]. Apart from these well-known technologies, other biomedical applications of light have been developed, such as photochemical tissue bonding [12,13,14], or light-activated drug delivery [15,16].

A critical aspect for the success of these technologies is the correct application of light to the target tissues. Usually, external laser sources are applied superficially, or as inserted optical fiber waveguides for access to deeper biological structures. Optical fiber waveguides are efficient devices for the delivery of light because of their small size, low cost, and efficiency. Although glass is biologically inert, these fibers are not appropriate for biological applications. The fibers are brittle, having sharp edges after rupture which can cause damage to surrounding tissues. Glass fibers are indeed very rigid and this mechanical unsuitability can damage surrounding soft tissue due to either fiber motion or natural body motions [17]. Implanted glass fibers are currently used to deliver light for treatment of malignant brain tumors [18], but this technique is limited to severe cases due to the risks outlined above [19]. As a consequence, flexible and biocompatible materials are needed as optical waveguides in clinical practice. In addition, additional surgery may be required to remove the fiber after use. An implantable and biodegradable waveguide would be of great interest to avoid this need.

A natural candidate in the search for a biocompatible fiber is silk fibroin (SF). This biomaterial shows notable qualities in terms of biocompatibility [20], mechanical resistance, and versatility of configuration in the field of tissue engineering; but, in addition, it also shows outstanding optical properties [21,22]. Its transparency to visible light, in the order of 98%, makes it suitable for the fabrication of scaffolds for cornea substitution [23].

Previously, regenerated silk optical waveguides have been printed on quartz without a cladding layer [24]. These waveguides had low loss propagation (0.25 and 0.81 dB/cm for the straight and curved waveguides, respectively, at 633 nm), an interesting property when the application requires light delivery focused in a point at the end of the fiber. However, as these waveguides were derived from the coagulation of regenerated aqueous fibroin, they were not free-standing and did not have the mechanical properties of a native fiber, limiting their utility for in vivo applications [24].

A few years later, Omenetto’s group presented an optical waveguide formed entirely of SF, for the delivery of light from the tip of the fiber to deep tissue [19]. The core of the waveguide was a long, narrow strip of silk film surrounded by a silk hydrogel. These SF waveguides are highly flexible but they present lack of mechanical strength, which complicates their medical uses. Recently, another approach has been presented by Nizamoglu et al., who prepared a planar, comb-shaped SF waveguide for photochemical tissue bonding (PTB) [14].

Our new approach, proposed in the present work, is the use of the ancient silkworm gut fibers (SGFs) as a light-diffusing fiber. This fiber, with a diameter of 300–500 µm, is obtained directly from the manual stretching of the silk gland after an acidification bath. This type of fiber was produced commercially as a surgical suture and fishing line, and was the origin, in the 19th century, of a flourishing industry in the region of Murcia in southeastern Spain. However, the development of nylon and other artificial fibers at a lower cost in the 1940s marked the complete disappearance of the SGF industry and the loss of the traditional know-how for its production [25,26].

Although SGFs are no longer competitive with similar synthetic fibers such nylon, because the costs of production on an industrial scale, they have other excellent characteristics. Among them we would highlight their outstanding mechanical properties. Although the values of tensile strength and strain at breaking found in SGFs are comparable to native silkworm silk (364 and 0.34 MPa, respectively), the much larger cross sectional area of SGFs implies that the forces that these fibers can sustain are four orders of magnitude larger than those sustained by native silkworm silk fibers. A maximum force of 68.64 N, corresponding to one of the curves presented, was measured for SGF [27].

As a consequence, SGF constitutes an excellent biomaterial for applications which require a structure for working under stress. However, after probing the optical properties of this fibroin configuration, it was found that SGF emits light laterally when illuminated through one of its ends acting as a “light-diffusing optical fiber” [28].

Henceforth, we present SGF as a biocompatible and bioabsorbable, light-diffusing optical fiber. The emission parameters of red and near-infrared coherent light are described and the stimulatory effect of red light on the metabolic activity of fibroblast cultures is measured. Although the concept of using fibroin to deliver light is not original [19,21,22], its configuration as a free-standing fiber is. This strong fiber allows better manipulation than previously described fibroin materials due to its superior mechanical behavior and tensile strength. The fibers can be tailored and inserted into tissue more easily than printed fibroin waveguides [19]. SGFs present also an easier fabrication process, which is an important advantage.

## 2. Results and Discussion

### 2.1. Fabrication of Silkworm Gut Fibers

**Figure 1:**
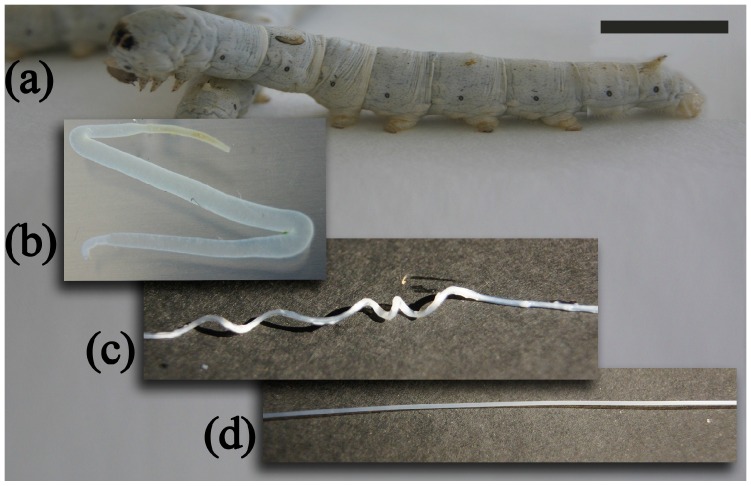
Production of silkworm gut fibers: (**a**) Caterpillar of a fifth instar silkworm; (**b**) Extracted glands immersed in a 2% acetic acid bath; (**c**) Part of the fiber forming a loop where the gland turns; (**d**) Dry fiber, cleaned of sericin and ready for use as a light-diffusing optical fiber. (Scale bar is 10 mm).

Following the preparation procedure described in the experimental section, manageable and firm SGFs ready for use were obtained. A graphical sequence of the process is showed in Figure 1. After the glands were stretched manually producing translucent fibers of ~0.5 mm in diameter. All SGFs were cleaned of debris by gentle manual rubbing prior to the diameter measurements. The fibers were dried, cut to the required length, and stored until characterization or use. Detailed information about the SGFs used in the study is presented in the Supplementary Material (Table S1). Although different silkworm races were used in order to compare the properties of different SGFs, in all cases, fibers of similar aspect were obtained, showing only macroscopic differences in their diameters.

### 2.2. Scanning Electron Microscopy

**Figure 2:**
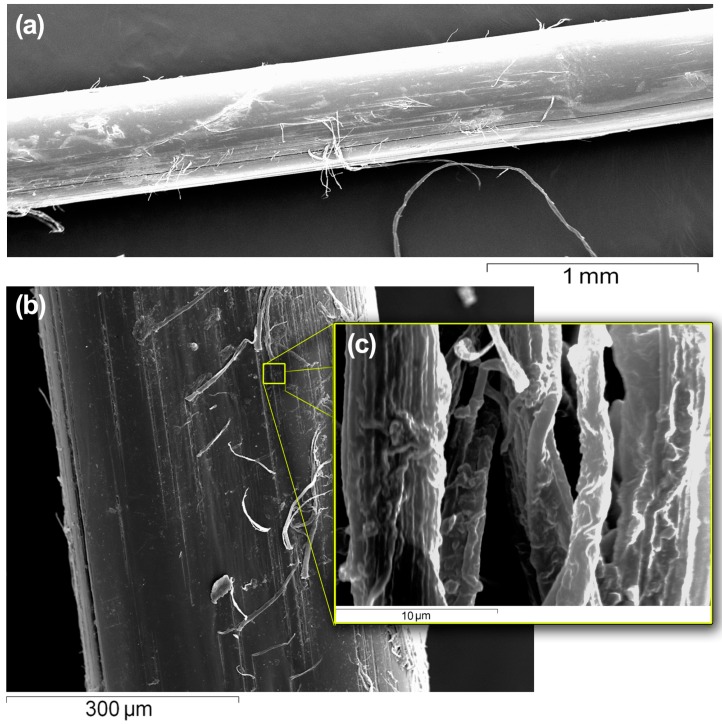
Scanning electron microscopy (SEM) images at different magnifications of a silkworm gut fiber (SGF) **5**, showing the irregularities on the surface of the fiber. (**a**) 30×; (**b**) 170×; (**c**) 5000×.

The scanning electron microscopy (SEM) pictures of the SGFs were used to visualize their topography and appearance. As can be observed in Figure 2, the fibers appeared cylindrical and uniform throughout their entire lengths at low magnification, but at high magnifications a roughened surface was apparent, on which microfilaments were appreciable. This roughness frustrates the total internal reflection phenomenon at the fiber-air interface, due to a scattering phenomenon, which promotes light diffusion out from the surface the fiber [29]. Diameter of the fibers observed in the SEM images corresponds with that measured with the microcaliper.

### 2.3. Attenuated Total Reflectance Fourier Transformed Infrared Spectroscopy (ATR-FTIR) Analysis

The conformation of the peptide chains of the silk fibroin determines the solubility and mechanical properties of the silk fibers [30]. Thus, infrared spectroscopy was selected to determine the conformation of SF in the SGFs. The analysis was focused on the region ranging from 1800 to 1200 cm^−1^, the most useful region for the analysis of SF amides (Figure S2b). The spectrum of the SGF **5** predominantly showed the characteristic peaks of β-sheets structures of the water insoluble Silk II. Amide regions I and II showed a strong signal at 1621 and 1517 cm^−1^, respectively, which are characteristic of β-sheets structures [30] similar to those presents in the spectrum of degummed SF fibers, measured as example of β-sheets structures (see Figure S2a). Those peaks coexisted with the broad absorption peak between 1537 and 1532 cm^−1^ (amide II), characteristic of random coil structures [31].

For comparison, the spectrum of a dry film of regenerated SF was also recorded (See Figure S2c). In this water-soluble state, the fibroin presents predominantly the random coil conformation. Amide regions I and II showed the characteristic peaks of the random coil at 1653–1645 cm^−1^ (amide Ι) and α-helix conformation at 1537–1532 cm^−1^ (amide ΙΙ), respectively [30,31,32,33,34].

The structural changes produced during the stretching of the concentrated SF solutions in the silk glands are reflected in the attenuated total reflectance fourier transformed infrared spectroscopy (ATR-FTIR) spectrum of the SGF. The peak of the amide I region shifted to lower wavenumbers as a consequence of the transformation from the random coil state in the silk gland to the highly crystalline β-sheet conformation in the SGF. This procedure of fast stretching of the guts limits the complete transition from random coil to β-sheet conformation and the material retained a small portion of random coil in its structure, as shown by a composed peak in the amide II region [31]. These results agree with our previously published for SGFs [27].

### 2.4. Characterization of Light Emission by Silkworm Gut Fiber (SGF)

Prior to the study of the stimulatory effect on cell cultures of red or near-infrared light delivered from SGF, characterization of the laterally emitted light from the fibers when illuminated with polychromatic light (multiple wavelengths) was performed. Using a halogen lamp as the light source (Osram 3000 K, 12 V, 20 W, GU5.3), which produces polychromatic warm light, the SGFs progressively turned from brilliant yellow to red in color down their lengths (See Figure S3). Thus, the SGF lost part of the confined light that faded along the fiber, showing a bathochromic shift of the maximum of the emission spectrum (towards longer wavelengths) along the fiber.

The SGFs did not show a definite cut-off wavelength (the minimum wavelength at which a particular fiber still acts as a single mode fiber) but shorter wavelengths did not produce total internal reflection inside the fiber and were not propagated efficiently. Only longer wavelengths were conducted further than the first few centimeters of the SGF, allowing diffusion of the red and near-infrared laser light from the fiber. To provide the proper amount of energy to the cell cultures, it is essential to know the energy radiated by the fibers for each light source. Indirect measurements of the irradiance emitted along the fiber were conducted for the SGFs using the experimental set-up described later in Section 3.4.

The results for different SGFs under study, illuminated with infrared and red radiation, showed that in all cases the measured irradiance (*E*) decreased exponentially with the length (*z*) traveled along the fiber, with the attenuation coefficient α as given by the fitting of an exponential curve to the experimental data (see Section 3.4), and being *E*_0_ the irradiance at the position of the first photodiode, *z* = 0. Detailed results of the fit are shown in Table S3.

For the red laser (RL) (λ = 650 nm), α values varied from 0.56 cm^−1^ for the SGF **5** to 1.03 cm^−1^ for the SGF **6**. For the near-infrared laser (NIRL) (λ = 808 nm), α ranged from 0.39 cm^−1^ for the SGF **5** to 0.73 cm^−1^ for the SGF **6**. From this we conclude that, in all cases, greater attenuation of red radiation by the SGFs than of infrared, which is consistent with the results from the analysis of the spectrum obtained with white light (Figure S3).

From the analysis of the fitted parameters, the SGF **5** (Italian polyhybrid (79 × 719) × (126 × 125)) was finally selected to perform the assay with cells because it showed a better performance in terms of coupling (the highest value of *E*_0_) and low attenuation along the fiber (the lowest value of α).

**Figure 3:**
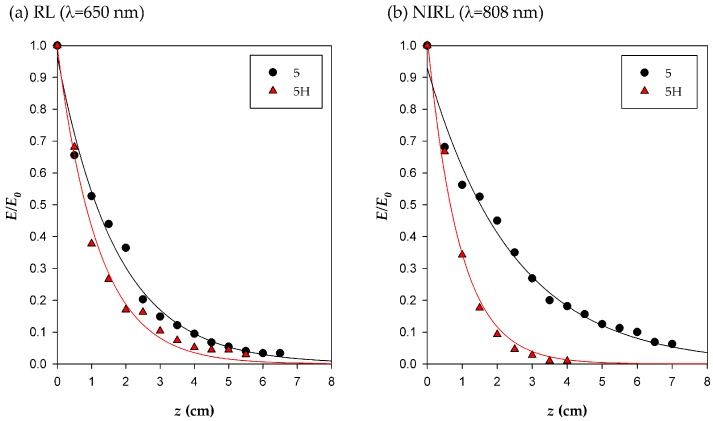
Exponential fit of normalized irradiances from the SGFs as a function of the distance to the coupling point *z* (cm). The SGFs tested in the experiments were dry (**5**) or hydrated (**5H**) fibers, using (**a**) Red laser (RL) or (**b**) Near-infrared laser (NIRL) as the light sources.

Hydrated SGFs **5** were also evaluated in order to study the effect of the light irradiated to the cells using these fibers in the same operational conditions as in the cell culture chamber. SGF **5H** was hydrated for 24 h before performing the irradiation experiment. The hydration of the fiber was also relevant to the amount of the emitted light, as can be observed in the different normalized curves for SGFs **5** and **5H** (Figure 3), also they showed different values of the attenuation coefficient (α) (Table S3). For comparative purposes, irradiances were normalized to the irradiance at *z* = 0 (*E/E*_0_).

The presence of water increased the values of the absorption phenomena recorded for the SGF with both light sources. As can be observed in Figure 3, Irradiance decay is intensified when SGF is hydrated with higher values of α. Differences are increased when near-infrared radiation is used, which is consistent with the results from the analysis of the spectrum obtained with the polychromatic light of a halogen lamp (Figure S3). By adjusting the power of the light source can be obtained irradiances with biological activity, like those previously described in literature [1].

### 2.5. Effect of Light Irradiated by SGF on Cells

**Figure 4:**
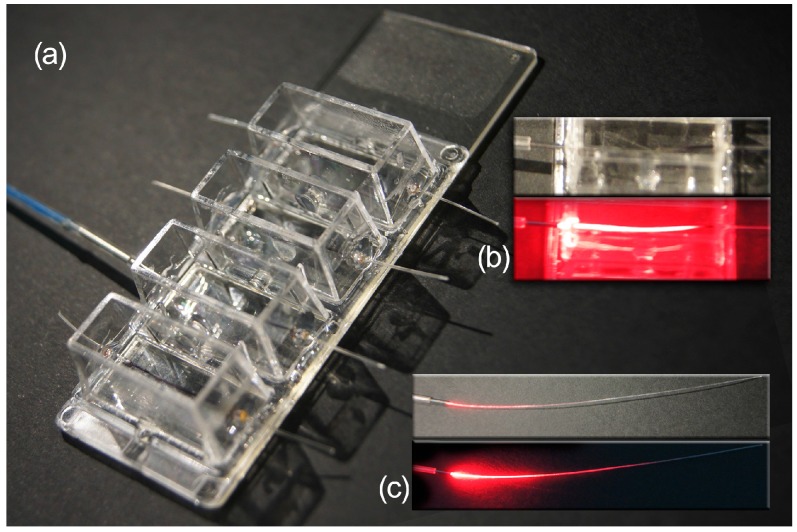
(**a**) Cell culture chambers set-up used for the red laser irradiation experiments; (**b**) Details of the RL light (λ = 650 nm) diffusion from the free-standing SGF **5** at ambient light and in darkness; (**c**) Details of the RL light diffusion from the SGF **5** inserted in the cell culture chamber at ambient light and in darkness.

In an effort to study the potential use of SGFs as biocompatible optic fibers to stimulate the proliferation of cells, L929 fibroblasts were seeded in culture chambers specifically designed to evaluate this effect. The experimental set-up appears in Figure 4.

**Figure 5:**
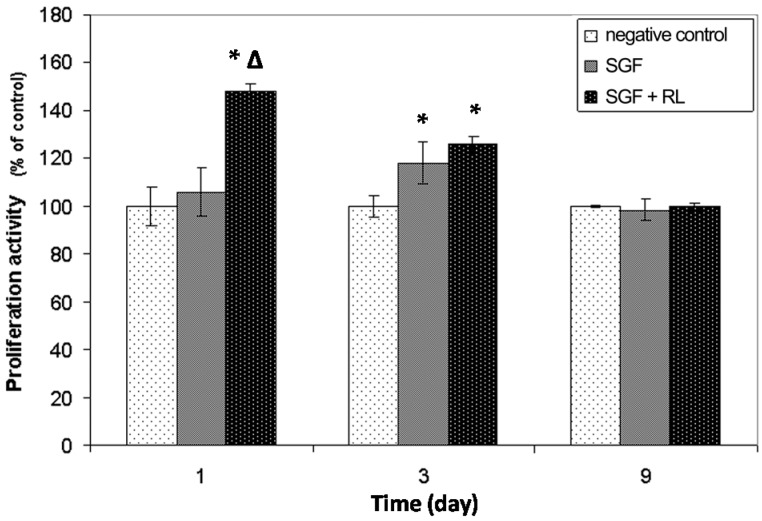
Proliferation activity of L929 cells cultured in different chambers, measured by the 3-[4,5-dimethylthiazol-2-yl]-2,5-diphenyl tetrazolium bromide (MTT) assay. “negative control”: 4-well standard chamber slides without any modification; “SGF”: chambers containing SGFs but without irradiation stimulus; “SGF + RL”: chambers containing fibers that irradiated RL light (λ = 650 nm). The data are presented as the percentage optical density (OD) obtained in relation to the negative control (mean ± sd (standard deviation)). (*) indicates statistical difference from the negative control and (Δ) indicates statistical difference from chambers with SGFs but without laser light irradiation (*p* < 0.05).

The RL was chosen as the light source because it is the one that has been described most fully in the literature [1]. In all cases, the irradiation was supplied to the cells with the SFG **5H**. The effect on the L929 cells induced by this irradiation was evaluated by the 3-[4,5-dimethylthiazol-2-yl]-2,5-diphenyl tetrazolium bromide (MTT) assay and the data are presented as the percentage optical density (OD) obtained in relation to the negative control (Figure 5).

The first MTT assay was performed one day after seeding and 1 h. after the irradiation of the chambers with red laser light. This test provided information related to the short-term activation of metabolism induced by the red laser light emitted by SGFs. Significant differences (Tukey, *p* < 0.05) were found between the OD values obtained in chambers that received irradiation and those of the other two treatments (chambers with SGFs which were not irradiated and negative controls). Irradiation of the fibroblast cultures resulted in a 48% increase in cell metabolism 5 h after receiving the stimulus (including the 4 h incubation with the MTT dye). This result can be explained as a consequence of the activation of the mitochondrial respiratory chain and the initiation of cellular signaling involved in proliferation, due to the irradiation with laser light. This has been stated by several authors and it is well known that low-level laser therapy can be used to promote proliferation of multiple cells [1].

**Figure 6:**
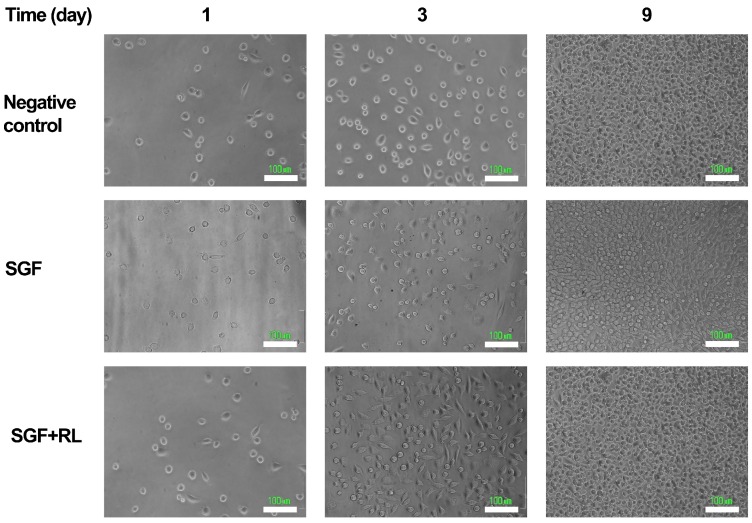
Photomicrographs showing L929 mouse fibroblast cultures seeded in different chambers: 1, 3, and 9 days after seeding. “Negative control” means standard cell culture chamber without any modification, “SGF” refers to chambers containing SGFs but without irradiation stimulus, and “SGF + RL” means chambers containing SGFs that received irradiation of RL light (λ = 650 nm). (Scale bar is 100 μm).

No differences were detected between negative control cultures and chambers containing non-irradiated SGFs in the culture medium (Tukey, *p* > 0.05), which means that one day after the seeding these materials were non-cytotoxic. The second MTT assay, developed three days after seeding and two days after the irradiation of the cultures, revealed significant differences in terms of OD between the two kinds of chamber containing the SGFs and the negative control (Tukey, *p* < 0.05). The average increase in the proliferation of the cells cultured in the irradiated chambers was 25.8%. A rise of 17.8% in non-irradiated chambers containing the SGFs was also detected; this could be the result of a stimulatory effect of the SF released by the material on the proliferation of fibroblasts. Finally, 9 days after seeding of the cells, the MTT results showed equal values of OD in all the treatments (Kruskal-Wallis, *p* > 0.05). This could have been due to a decrease in the stimulatory effect of the red light irradiation in the long-term and also to an inhibitory effect on cell growth at the confluence. On the first day of the study the appearance and confluence of the cells observed by microscopy (Figure 6) were similar in all the treatments, meaning that the increase in OD detected by MTT staining was directly related with the rise in the metabolic (mitochondrial) activity and not with the number of cells in the culture.

The study by microscopy of the samples three and nine days after seeding confirmed the results revealed by the MTT assay. Three days after seeding, greater confluence and expansion of the L929 fibroblasts was observed, mainly in “SGF + RL” culture chambers, but also in “SGF” chambers, where the cell culture was not irradiated. At the end of the experiment (nine days), all the chambers presented similar cellular confluence and morphology.

**Figure 7:**
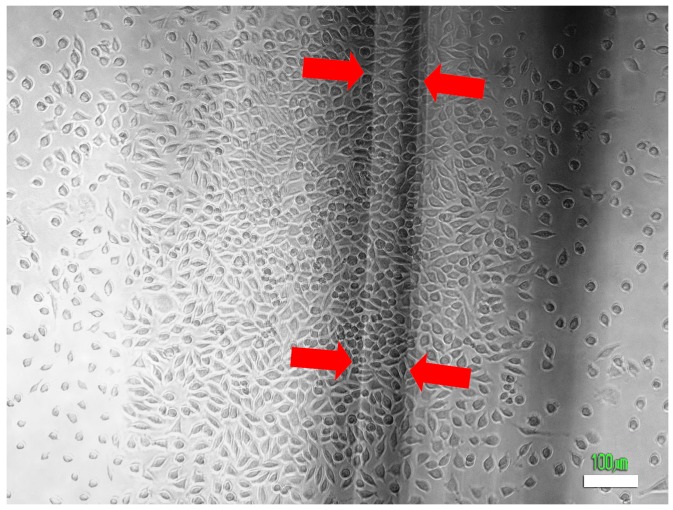
Photomicrograph showing the view of the bottom of the culture chamber with L929 mouse fibroblasts two days after irradiation with RL light (λ = 650 nm) from SGF **5**. The arrows demarcate the position of the fiber above the plane of the cells. It can be observed that the cell density is higher below the fiber than on the rest of the culture surface. (Scale bar is 100 μm).

The cell density was higher below the irradiated SGFs, a result of a more effective incidence of red laser light (Figure 7). A stimulatory effect due to a local increase in temperature along the fiber was discounted because the measured temperature of the chambers remained almost constant during the experiments.

## 3. Experimental Section

### 3.1. Fabrication of Silkworm Gut Fibers

Larvae of the fifth instar of *Bombyx mori* L. were reared on mulberry leaves. Different *B. mori* breeds were used in order to compare the properties of different silks (Table S1). The fabrication followed the procedure described in previous work developed by our research group [27]. Briefly, the process started with the anesthesia of the larvae, by maintaining them at 4 °C. The head of each larva was cut off with a razor blade and the two silk glands were extruded by internal pressure. The glands were washed in water and transferred to a bath of 2% acetic acid for 2 min (Figure 1). After that, the glands were stretched manually from each end, to their maximum length of about 40–50 cm. This resulted in a translucent fiber, ~0.5 mm in diameter and covered by debris composed of cells and sericin, which was removed by washing in water and manual rubbing. The clean fiber was dried, cut to the required length, and stored. The diameter of each SGF was measured with a microcaliper (Mitutoyo Absolute Digimatic 200 mm/8 Caliper-500-197-30, Mitutoyo, Japan), with a resolution of ±0.01 mm and an accuracy of ±0.02 mm.

### 3.2. Scanning Electron Microscopy

A piece of ~1 cm length of SGF **5** was fixed on an aluminum stub using double sided adhesive carbon tape, coated with gold under vacuum by an auto fine coater, and examined at different magnifications using a scanning electron microscope (JSM-6060, JEOL Ltd., Tokyo, Japan) at 15 kV.

### 3.3. ATR-FTIR Analysis

Attenuated total reflectance fourier transformed infrared spectroscopy (ATR-FTIR) was used to analyze the structural conformation of SF after processing the SGFs. A dry sample of SGF **5**, a sample of degummed SF fibers obtained from silk cocoons [32], and a dry film of regenerated SF (after dissolution in LiBr 9.3 M and further dialysis) [33], were used directly for ATR-FTR measurements without further manipulation. Degummed SF was selected as a reference for the β-sheet structure of SF and dry film of regenerated SF as an example of a predominantly random coil and α-helix conformation. Each spectrum was acquired on a spectrometer (Nicolet™ iS™ 5 FT-IR spectrometer, Thermo Electron Scientific Instruments LLC, Madison, WI, USA), equipped with an ATR accessory (iD™ 5, Thermo Electron Scientific Instruments LLC) controlled with OMNIC Specta software (Ver. 9.3.30, Thermo Electron Scientific Instruments LLC), measuring in absorbance mode with a resolution of 4 cm^−1^, a spectral range of 4000–550 cm^−1^, and 64 scans. The analysis was finally focused in the range of 1800–1200 cm^−1^, the most informative for the IR spectra of SF (amide I and II regions). Vibrational band assignments were based on the data summarized by Hu et al. [30].

### 3.4. Characterization of Light Emission by SGF

**Figure 8:**
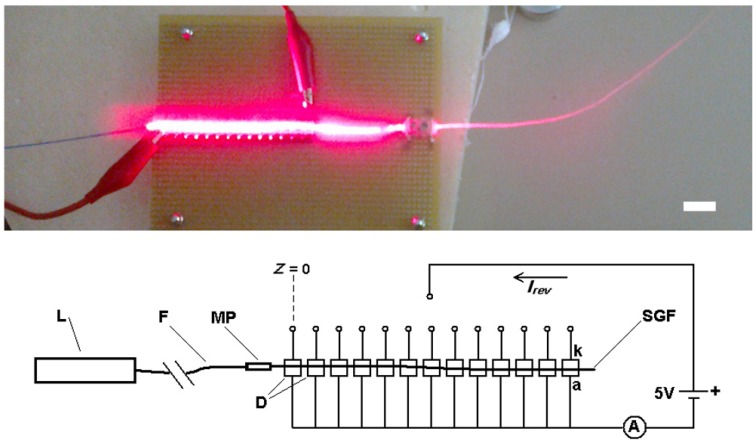
Experimental set-up for irradiance measurements: L, laser; F, optic glass fiber; MP, metal pipe; D, photodiodes; SGF, silkworm gut fiber; A, amperemeter; a, diode anode; and k, diode cathode. (Scale bar is 20 mm).

As stated above, the SGF behaves like a light-diffusing fiber [29]. The irradiated light (*E*) was indirectly measured by using a reverse-polarized 15-photodiode linear array (Figure 8).

The photodiodes were separated by a distance of 5 mm between their centers, with the fiber leaning directly on the diodes. The laser light was conducted through an optic glass fiber (0.5 mm glass core diameter) coupled face-to-face to one suitably polished end of the SGF by a metal pipe connector (internal diameter of 0.5 mm, 20 mm in length) to minimize the attenuation that occurs for transverse displacement, angular misalignment, or longitudinal spacing between the optic glass fiber and SGF.

This low-cost experimental set-up for the in situ characterization of the irradiance of SGF can be easily removed and the coupled fibers are ready to be used for the in vitro experiment after characterization and optimization of coupling.

Two lasers of 200 mW were used as light sources: a red laser (RL), λ = 650 nm (model H650L) and a near-infrared laser (NIRL), λ = 808 nm (model 301-LM) (see Table S2 for manufacturer specifications). The used photodiodes (Silicon PIN Photodiode BPW34, Vishay Semiconductors, Shelton, CT, USA) present high sensitivity to visible and near infrared radiation. Their radiant sensitive area was 7.5 mm^2^. The light source was pulsed in order to avoid thermal damage to the fiber. This was achieved with the on/off switch of the power supply connected to the laser (one minute “ON” followed by one minute “OFF”). The irradiance is related to the incident light on the photodiodes, and was determined from the measurement of its reverse current, *I*_inv_. The manufacturer of the photodiodes provided a technical data sheet with a graph of *I*_inv_ as a function of *E* at a wavelength of 950 nm, and the curve for the relative spectral sensitivity, *S*, as a function of wavelength, λ (Figure S1).

The irradiance is determined at the working wavelength as:

(1) $$E(\lambda )=\frac{I_{\text{inv}}S(\lambda )}{R}$$

where *R* is the *I*_inv_ to *E* ratio at a wavelength of 950 nm.

The experimental data were fitted to the exponential curve (Sigma Plot Software V. 11.0, Systat Software Inc., San Jose, CA, USA)) (see Section 2.4.):

(2) $$E(z)=E_{0}e^{-\alpha z}$$

where “α” is the attenuation coefficient of the light emission from the fiber and “*E*_0_“ is the irradiance at the position of the first photodiode (*z* = 0), with “*z*” being the distance from it to each photodiode along the fiber. In the SGFs, the coefficient “α” is mainly related to the scattering and absorption phenomena along the fibers.

The energy in the desired area is calculated (see Appendix A of the Supplementary Material for further details) and is controlled using pulsed radiation.

### 3.5. Effect of Light Irradiated by SGF on Cells

#### 3.5.1. Cell Culture

In order to test the potential use of SGFs as light-diffusing fibers for laser stimulation, in vitro studies were performed using the murine fibroblasts L929 cell line (European Collection of Authenticated Cell Cultures (ECACC), Catalogue No.: 85011425). The L929 cells were chosen for the cell culture studies as they are highly stable, fast growing, and commonly used for cytotoxicity and biocompatibility experiments. All the chemicals for the cell culture were purchased from Sigma-Aldrich (St. Louis, MO, USA) and the cell culture chambers and flasks were provided by Nunc (Roskilde, Denmark). The cells were cultured in flasks (75 cm^2^), in Dulbecco's Modified Eagle Medium (DMEM) medium supplemented with 10% fetal bovine serum (FBS), 100 U/mL penicillin, 100 μg/mL streptomycin, and 0.1 mM non-essential amino acids, in a humidified incubator with 5% CO_2_ at 37 °C. When the cells reached 80% confluence, they were detached using 0.25% trypsin, 1 mM ethylenediaminetetraacetic acid (EDTA) and subcultured at a seeding density of 5000 cells/cm^2^ in new flasks. Cell proliferation and cell number were routinely determined by 3-[4,5-dimethylthiazol-2-yl]-2,5-diphenyl tetrazolium bromide (MTT) and standard trypan blue staining, respectively, and the culture medium was replaced every three days.

#### 3.5.2. Irradiation of the Cell Culture Assembly

Lab-Tek^®^ 4-well chamber slides (mounted on Permanox^®^) (Thermo Fischer Scientific Inc. Rochester, NY, USA) were modified to perform the irradiation experiments. Both sides of each well were perforated in order to introduce one piece of SGF (4 cm in length and 340 ± 40 µm in diameter) that crossed the well 3 mm above the culture surface of the slide. Then, the holes were sealed with DOW CORNING 3140 RTV (Dow Corning Corp. Midland, MI, USA) coating in order to prevent leakage of the culture medium (Figure 5). The L929 cells were selected to probe the potential stimulatory effect of the irradiated light on the proliferation of the cell cultures. The experimental set-up included as “controls” chambers without SGFs, chambers with an SGF but without light stimulus, and chambers irradiated with red laser light from an SGF. The chambers were sterilized with 70% ethanol for 10 min and rinsed with sterile MilliQ water before the seeding. Then, L929 cells were cultured at a density of 5000 cells/cm^2^ in DMEM medium (same composition and conditions as described previously) and seeded. Twenty-four hours after the seeding, the culture medium was replaced by phosphate buffer saline (PBS) (Sigma-Aldrich) 1×, pH 7.4, and the irradiation of the cells was carried out in darkness, without influence of light other than that of the laser.

According to the irradiance properties of the SGF **5H** described in the Section 2.4. and the design of the culture chambers, the average value of the irradiance received on the culture surface of each well was estimated at 0.64 mW/cm^2^, corresponding to a total power of 1.03 mW. After a total exposure time to laser irradiation of 330 s per well, the average value of the energy density received by the cells was 0.21 J/cm^2^ (see Appendix A of the Supplementary Material for detailed calculations). This value is within the range proposed by other authors who used different set-ups and fibroblasts from a wide variety of sources; these data are summarized by AlGhamdi et al. [1].

#### 3.5.3. Cell Proliferation Assay

The proliferation of cells was evaluated by using MTT (Sigma-Aldrich). The MTT is reduced to purple formazan derivatives in living cells by means of cellular (mitochondrial) respiration. Therefore, from this assay, the cellular metabolic rate and viability and/or proliferation of cell cultures can be inferred [35]. The MTT experiments were performed one, three, and nine days after seeding. All the treatments were performed in triplicate. The culture medium was removed and 500 μL of MTT dye solution (1 mg/mL in DMEM without phenol red) were added to each well and incubated for 4 h at 37 °C and 5% CO_2_. Then, the MTT solution was removed and the formazan crystals were solubilized with 200 μL of dimethyl sulfoxide (DMSO) (Sigma-Aldrich) per well. The chambers were vigorously shaken for 5 min to dissolve the reacted dye. After transfer to a 96-well plate, the absorbance of 100 μL of DMSO per well (containing solubilized formazan) was read on a microplate reader (BMG Fluostar Galaxy, Ortenberg, Germany) at 570 nm. Unreacted MTT was measured as the absorbance at 690 nm [35]. The proliferative activity measured by the MTT assay was expressed as the percentage of the optical density value obtained in each sample, relative to the “control group” chambers (without SGFs).

#### 3.5.4. Microscopy

Cells were visualized with an inverted microscope (Nikon Eclipse TE2000-U, Nikon Instruments Inc. Melville, NY, USA), equipped with a digital camera (Nikon DS-5M,, Nikon Instruments Inc. Melville, NY, USA). Images of the same portion of the tissue culture surfaces were captured 1, 3, and 9 days after seeding in order to illustrate the appearance and confluence of cells at the times when the MTT assay was performed.

### 3.6. Statistical Analysis

The data are presented as mean ± sd (standard deviation). These data were calculated from three samples per condition. If the assumptions of normality (Kolmogorov-Smirnov, *p* > 0.05) and homocedasticity (Levene, *p* > 0.05) were met, the statistical significance was determined using the parametric tests of Tukey (*p* < 0.05) and ANOVA (*p* < 0.05) for comparisons of two or more groups, respectively. If these requirements were not satisfied, the Kruskal-Wallis test (*p* < 0.05) was used to determine these differences. For statistical analyses, SPSS software was used.

## 4. Conclusions

This work describes the use of an implantable, biocompatible, and biodegradable, natural silkworm gut fiber (SGF) of *B. mori*, for light delivery in deep tissues. The SGF behaves like a light-diffusing fiber, which is desirable when the objective is to activate a significant area of an internal tissue instead of a localized point.

Moreover, a low-cost experimental set-up for in situ characterization of the irradiance from the SGFs coupled to a glass fiber has been also developed. After the measurements have been performed, the actual irradiance is easily calculated. Then, detectors are easily detached from the fibers and SGFs coupled to the glass fiber are ready to be used for the in vitro experiment. Thus it is possible to adjust the dose of light delivered to the cells by varying the lighting time.

The SGF performs like a light-diffusing fiber, due to the natural irregularities of the surface and the scattering phenomena inside the fiber. In this sense, it is not properly an optical fiber, whose utility is to carry light efficiently from one point to another. The SGF, instead, maximizes the light diffused along the fiber; this is desirable when the objective is to affect a considerably higher number of cells.

The work also describes the stimulatory effect on cell proliferation of the light emitted by the fiber. The average energy density received by the cells after a total exposure time to irradiation was estimated at 0.21 J/cm^2^. The results are similar to those stated by other authors, who reported increases in proliferation or in the number of viable cells due to stimulation with red laser light of different cell cultures as human skin fibroblasts [3,6], human gingival fibroblasts [2], human dental pulp stem cells [4], and epithelial cells [7] among others. Our in vitro study is the first step towards the use of a novel, free-standing biomaterial with a wide variety of potential applications in phototherapy and wound healing [14,36].

Another advantage of SGF is the possibility to perform chemical modifications of its surface with small molecules or proteins, offered by the fibroin, by means of several described techniques [37,38]. This modification could also be made with metallic particles or conducting polymers and, in this way, the fiber would acquire electric conductivity, constituting an optrode for dual signaling. On the other hand, it could be covered with a polymer encapsulating photoactive molecules, allowing applications in the fields of photodynamic therapy or light-activated drug delivery [35]. Finally, the outstanding properties of SGF, in terms of mechanical resistance and facility of fabrication and use, make it an excellent alternative to previously described regenerated silk fibroin optical waveguides.

## Supplementary Materials

Supplementary materials can be found at http://www.mdpi.com/1422-0067/17/7/1142/s1.
