# Supplementary material for: Silkworm Gut Fiber of Bombyx mori as an Implantable and Biocompatible Light-Diffusing Fiber
Source: Int J Mol Sci. 2016 Jul 16;17(7):1142. doi: 10.3390/ijms17071142 (PMC4964515; doi:10.3390/ijms17071142)
Supplement: Supplementary file 1 [file ijms-17-01142-s001.pdf]

Supplementary Materials: Silkworm Gut Fiber of *Bombyx mori* as an Implantable and Biocompatible Light-Diffusing Fiber

Jose Luis Cenis, Salvador D. Aznar-Cervantes, Antonio Abel Lozano-Pérez, Marta Rojo, Juan Muñoz, Luis Meseguer-Olmo and Aurelio Arenas

Table S1. List of silkworm gut fibers (SGFs) used in the study and their characteristics.

Reference	SGF Diameter (mm) †	*B. mori* Races	Cocoon Color
**1**	0.32 ± 0.05	Sierra Morena	Green
**2**	0.27 ± 0.04	Murcian Gold	Yellow
**3**	0.34 ± 0.06	Murcian Gold	Yellow
**4**	0.42 ± 0.06	Murcian White	White
**5**	0.33 ± 0.08	Italian Polyhybrid *	White
**5H** §	0.34 ± 0.08	Italian Polyhybrid *	White
**6**	0.44 ± 0.07	Italian Polyhybrid *	White

† Mean ± sd (standard deviation) of 6 SGFs obtained from different caterpillars of each *B. mori* silkworm races studied; * The SGFs **5** and **6** were produced from the same Italian polyhybrid breed (79 × 719) × (126 × 125) provided for Silvia Capelozza, CRA-API Unità di Ricerca di Apibachicoltura, in Padova (Italy), but differ in thickness; § The SGFs **5** and **5H** were produced from the same Italian polyhybrid (79 × 719) × (126 × 125) but differ in water content (**5H** were immersed in water at least 24 h prior to the measurements).

Table S2. Laser specifications.

Laser	H650L	LM-301
Wavelength	650 nm	808 nm
Size	180 mm × Φ23 mm	140 mm × Φ20 mm
Output power	200 mW	200 mW
Working mode	CW	CW
Working voltage	3.7 V	3.7 V
Divergence	<2.0 rad	<2.0 rad
Laser class	3B	3A

Table S3. Values obtained after the adjustment of the experimental data to the exponential curve of the radiant flux received on the surface of the cell chamber from different SGFs when are illuminated with red laser (RL) or near-infrared laser (NIRL).

SGF	RL (λ = 650 nm)		NIRL (λ = 808 nm)	
	α (cm^{-1}) †	E_0 (mW/cm^2) §	α (cm^{-1})	E_0 (mW/cm^2)
**1**	0.84 ± 0.02 (3%)	31 ± 2 (6%)	0.53 ± 0.02 (3%)	23 ± 1 (4%)
**2**	0.74 ± 0.02 (3%)	28 ± 2 (7%)	0.48 ± 0.03 (7%)	15 ± 2 (13%)
**3**	1.04 ± 0.08 (7%)	13 ± 2 (15%)	0.53 ± 0.03 (6%)	20 ± 2 (10%)
**4**	0.80 ± 0.01 (2%)	41 ± 2 (5%)	0.46 ±0.01 (3%)	38 ± 2 (5%)
**5**	0.56 ± 0.01 (2%)	47 ± 2 (4%)	0.39 ±0.01 (3%)	32 ± 1 (3%)
**5H**	1.35 ± 0.01 (1%)	50 ± 4 (8%)	0.68 ±0.03 (4%)	25 ± 2 (8%)
**6**	1.00 ± 0.02 (2%)	85 ± 4 (5%)	0.73 ±0.01 (2%)	60 ± 3 (5%)

† α, attenuation coefficient; § E_0, Irradiance at $z = 0$. Values presented as Mean ± sd (%CV). $n \geq 3$.

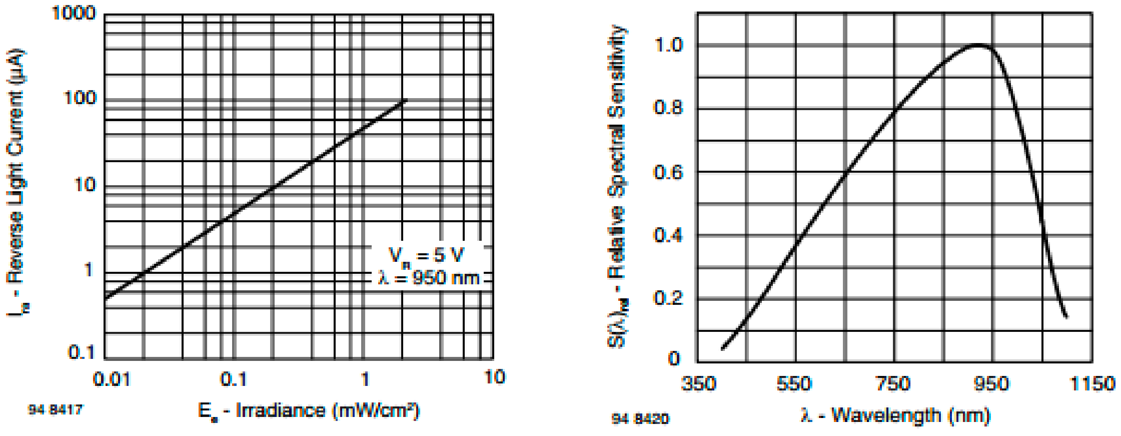

(**a**) (**b**)

Figure S1. Curves obtained from the datasheet provided by the photodiode manufacturer, at www.vishay.com. (**a**) Graph of I_{rev} versus irradiance, *E*, at 950 nm; (**b**) Graph of the relative spectral sensitivity, *S*, versus wavelength, *λ*.

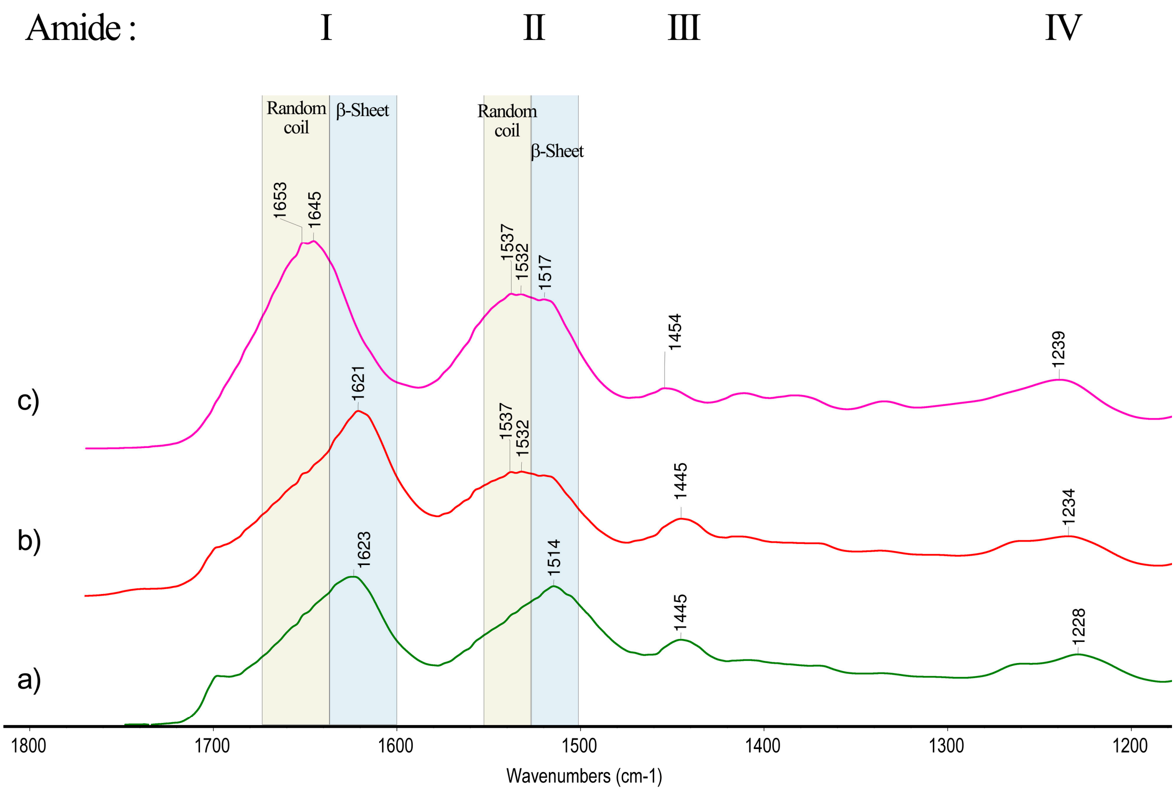

Figure S2. Region 1800–1200 cm^{-1} of the attenuated total reflectance fourier transformed infrared spectroscopy (ATR-FTIR) spectra of samples of silk fibroin. (a) Degummed silk fibroin (SF) obtained from white cocoons; (b) SGF 5 and (c) Dry SF film after dissolution in 9.3 M LiBr and dialysis.

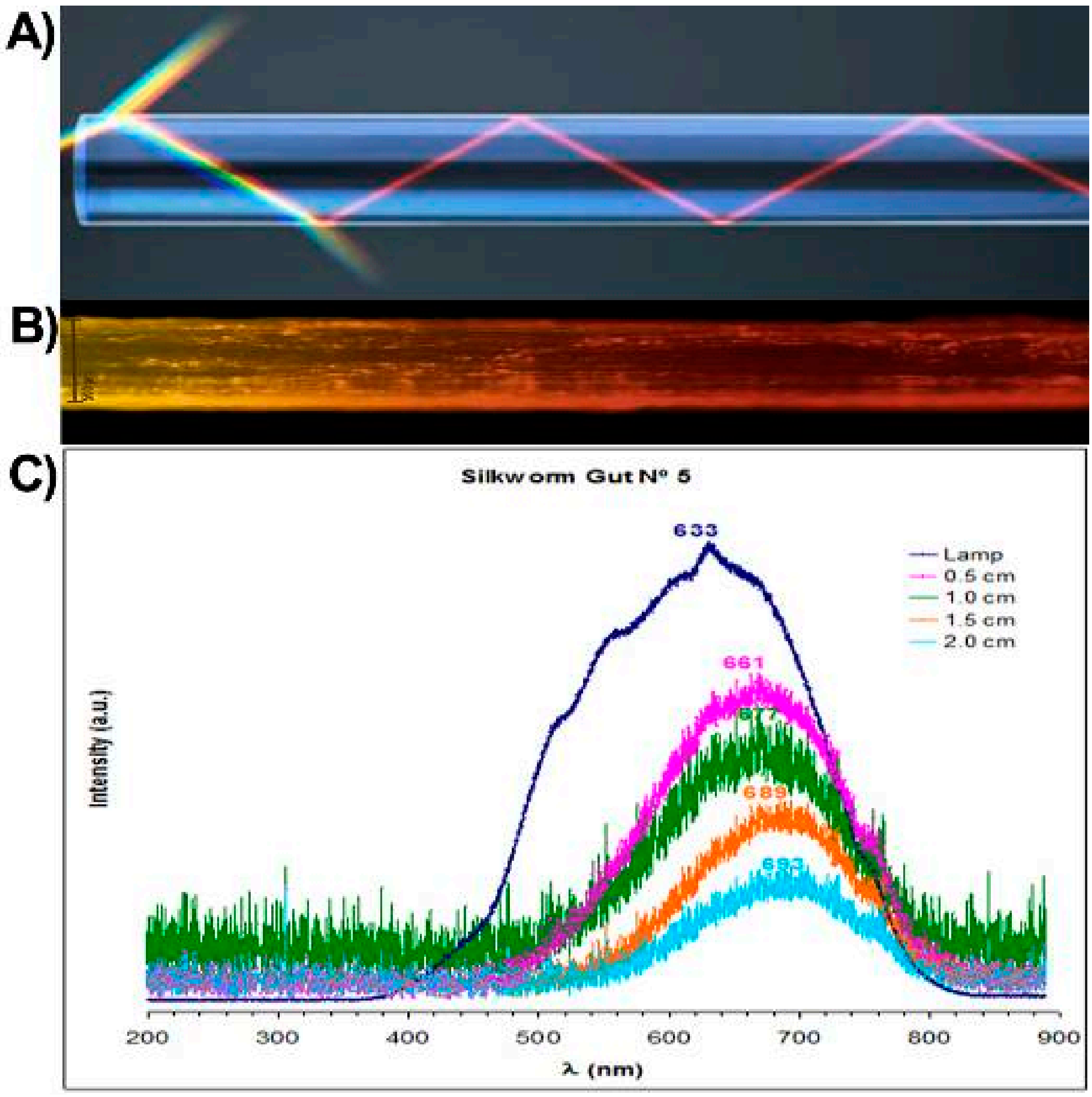

Figure S3. (**A**) Scheme of the cutoff wavelengths of the incident light produced in a Corning F101 optic waveguide (adapted from Corning with permission); (**B**) Micrograph of SGF 5 when illuminated with an OSRAM halogen lamp (Osram 3000 K, 12 V, 20 W, GU5.3) in a set-up analogous to that described in Figure 2. (Scale bar is 500 μm); (**C**) Emission spectra of diffused light from SGF 5, measured with an Ocean Optics USB 4000 portable spectrophotometer, as a function of the distance to the coupling with the lamp, in the same conditions as in N). The spectrophotometer was set at 1 cm above the fiber in all cases.

Appendix A. Calculation of the Energy Emitted by the Silkworm Gut

We proceed from equation of Irradiance (E) of a silkworm gut fiber (SGF) of radius r_g, just on the lateral surface ($r = r_g$), as a function of distance (z) along the SGF:

$$E(z) = E_0 e^{-\alpha z} \ (mW/cm^2) \tag{1}$$

where α is the attenuation coefficient related with the decay of radiated energy along the SGF and E_0 is the irradiance at $z = 0$, both given by the fit of an exponential curve to the experimental data. The irradiance at any other distance, r, from the fiber axis is:

$$E(r,z) = E_0 \frac{r_g}{r} e^{-\alpha z} \tag{2}$$

The total power incident on the surface of the cell culture chamber, P_c, will be equal to the power radiated through the lateral wall of the SGF that reaching the culture surface (S_c), which extends between z_i and z_f, over an angle ϕ (see Figure S4):

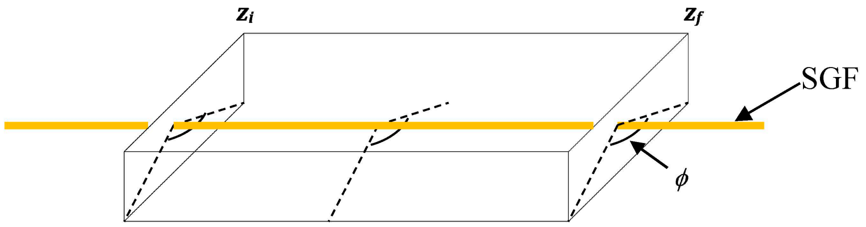

Figure S4. Cell culture chamber crossed by the SGF.

$$P_c = E_0 \cdot \int_{z_i}^{z_f} e^{-\alpha z} \cdot dz \cdot r_g \cdot \int_0^{\phi} d\phi \quad (mW) \tag{3}$$

In our case, the culture surface S_c = 1.6 cm², the angle ϕ = 1.74 rad, z_i = 0 cm and z_f =2 cm. For the SGF **5H**, E_0 = 50 mW/cm², α = 1.35 cm⁻¹, r_g = 0.17 mm. By performing the calculations, it is obtained that the power received on the culture surface is 1.03 mW.

Consequently, the average Irradiance on the culture surface P_c/S_c = 0.64 mW/cm². The average Energy received after a total exposure time Δt =330 s is 0.34 J and the average energy density received by the cells in the cell chamber surface is 0.21 J/cm².
